# Trunk appearance perception scale (TAPS) discrepancy between adolescents with idiopathic scoliosis and their parents influences HRQL

**DOI:** 10.1186/1748-7161-8-S1-O55

**Published:** 2013-06-03

**Authors:** M Rigo, E D'Agata, M Jelacic

**Affiliations:** 1 Institut Elena Salvà, Barcelona, Spain; 2 Fund. Hosp. Univers. Vall D'Hebron - Institut de Recerca. Barcelona, Spain

## Background

The Trunk Appearance Perception Scale (TAPS) is a valid instrument to assess self-perception of trunk deformity [1]. The SRS-22 has been widely used to measure Health Related Quality of Life in scoliosis population, but it is not clear which factors can influence its final score [2]. Children and parents can perceive trunk deformity differently, and discrepancy can be assessed with a coefficient of discrepancy (CD= TAPS major - TAPS minor x 100/ TAPS major). We already found that the CD influences the SRS-22 score. However, the previous study included patients older than 18 years, both genders, and results were not reported according to age [3].

## Aim

The aim of this new study is to confirm previous results, in a larger sample size, including only girls from 10 to 18 years of age (adolescents).

## Methods

Prospective study including 107 girls diagnosed with idiopathic scoliosis (treated and untreated), attending the clinic with their parents. Mean age 14.4 years. Mean Cobb angle 33.5° (10-75). All patients completed the SRS-22 and the TAPS. Parents completed the TAPS assessing trunk deformity of their children. A coefficient of discrepancy (TAPS-CD) was defined. Statistical analysis (SPSS) was made to compare TAPS, TAPS-CD and SRS-22.

## Results

Results confirmed previous findings. A significant correlation was found between patients' TAPS and SRS-subtotal, pain, self-image and mental health and between parents' TAPS, function and treatment satisfaction in the SRS-22. Parents' TAPS did not correlate with the Cobb angle, but with SRS-subtotal of the girls' self-image, pain and mental health. TAPS CD showed a significant correlation with SRS-Subtotal, self-image and mental health. Two groups were created according to the SRS-22 score. Patients with lower score in the SRS-22 showed a higher TAPS-CD (P<.05). These results were not different in girls aged 10 to 14 and girls aged 14 to 18.

## Conclusion

Discrepancy in “perception of trunk deformity” between adolescent girls and parents influence the SRS-22. Whether such a discrepancy is a factor for a lower quality of life, later, during adult life, is an open question.
